# Medical Electricity

**Published:** 1904-05-28

**Authors:** 


### MEDICAL ELECTRICITY.

That the *x*-ray tube is of service in the more complete definition of diseased lung in phthisis is pointed out by Brock and Stanley Green[1] in a paper giving skiagraphic representations of phthisical cases before and after sanatorium treatment. They have now had a number of cases to base their remarks upon, and declare that :—(1) in no single case where the physical signs have pointed to disease have the rays failed to detect mischief ; (2) in some cases where physical signs have been absent the rays have shown deposit in the lungs, and in these cases physical signs have subsequently been detected ; (3) the early diagnosis is certainly helped ; (4) that the extent of the disease is in many cases shown to be greater than the physician thinks ; (5) that the progress and results of treatment can be watched with greater accuracy.

Chisholm Williams[2] has not had reason to be disappointed with the high frequency currents as used in the treatment of phthisis. In 1901 he recorded 43 cases under treatment, and now of these three have died, of pneumonia, of tuberculous kidney, and of lardaceous disease. He advises that the apparatus be of the most powerful available. In tuberculosis of other parts, joints, etc, the best results have been obtained by general electrification combined with a high vacuum electrode used from the resonator, or the ordinary *x*-ray discharge. Cases of old-standing tuberculous lesions he states to be very amenable to treatment. In the treatment of lupus he finds the *x*-ray tube as reliable and to produce as good results as the light treatment. He urges the use of the high vacuum electrodes with a vacuum high enough to produce a fluorescence on an *x*-ray screen. Also the patient should receive on the condensation couch as much as 350 milliampères and upwards. It has been suggested from several quarters that the action of the *x*-rays upon tubercular lesions may render the tuberculosis more diffuse, and that phthisis might ensue upon lupus, and peritonitis upon glands, etc., but in the opinion of Jamieson[3] and others this is probably no more than a mere coincidence.

In opening the discussion upon the subject of electro-therapeutics in the treatment of malignant disease, at the annual meeting Lewis Jones[4] raises numerous questions of the first importance which demand solution before much advance can be expected. Notably is this the case in the matter of what kind of rays are of most advantage—whether the "*x*-rays," "cathode rays," or a combination of the two. He personally recommends the use of a "medium" tube, and prefers to operate with the anti-cathode red hot. He avoids dermatitis by arranging the exposures suitably, and continues the treatment for three, four, or five months.

In continuing this discussion, Hall Edwards related a case of epithelioma of the nose which under the *x*-rays showed very little tendency to improvement until potassium iodide was administered, when the affection rapidly disappeared. The drug had been previously administered without any improvement. Chisholm Williams recommends the use of the high frequency currents, administered by means of a high vacuum electrode to the affected part. This, in his hands, has proved more effective than the ordinary *x*-ray tube. Manders advocates the use of a blue-coloured (cobalt-glass) vacuum electrode. Lovell Drage considers that success in the treatment of cancer will be obtained with a combination of electrical treatment and the use of some drug such as the subcutaneous injection of a 10 per cent. glycerine solution of sodium cinnamate, which produces considerable leucocytosis.

Allan Jamieson[5] in speaking of the employment of hard or soft tubes in *x*-ray work, states that he has found that weather affects the rays materially, *e g.*, on cold raw days reactions more readily occur. He has had some success with the *x*-rays alone in lupus in the interior of the nose, but rather leans to the use of the ordinary methods prior to the *x*-ray treatment. He has abandoned the use of adrenalin as an adjuvant to the light treatment as "the exaltation in the effect was not at all commensurate with the expense." In rodent ulcer he has had some success, when the periosteum and bone have been involved. At the same meeting Dawson Turner showed a case of scirrhus erythema following removal of breast, which had been removed entirely by means of 46 exposures of 4 mins. each (*x*-ray). (2) A case of fibro-sarcoma of the frontal bone, the swelling of which had nearly occluded the right eye. After 11 exposures of 4 minutes the swelling had greatly diminished and the patient could see easily. A case of recurrent scirrhus in which 26 exposures were sufficient to cause the nodular and glandular swellings to disappear. And a case of recurrent sarcoma which had so improved after 48 exposures that the man had returned to work.

With regard to the further reported cases of cancer treated with the *x*-rays, Wild[6] gives notes of 10 cases of epithelioma, inoperable or recurrent, in two of which temporary subsidence occurred with recrudescence of the disease later, and in the rest no benefit was derived other than diminution of pain and lessening of discharge. He has grave doubts as to the prophylactic value of the *x*-rays in preventing recurrence after operation for cancer. He reports also eight cases of advanced scirrhus of breast and six recurrent after one or more operations. With one exception there was relief from pain, even also when the rays were applied with this object to a secondary deposit in the liver. In three cases there was considerable temporary improvement. The rate of progress was certainly checked. No result occurred in an inoperable primary growth in the parotid.

A remarkable case of spleno-medullary leucæmia has been treated by means of the x-rays by Nicholas Senn[7] with complete success. The patient, a Jewess, aged 29, had an enormously enlarged spleen which nearly reached the pubes below and extended two inches beyond the median line on a level with the umbilicus. Under drug treatment no improvement resulted, and as a last resort x-rays were tried. The spleen, the lower end of sternum, and the epiphysial extremities of the long bones were exposed to the rays daily for from 10 to 20 minutes. At the commencement, February 3rd, the blood examination yielded the following result: Erythrocytes, 3,500,000; leucocytes, myelocytes, and eosinophiles, 64,800; hæmoglobin, 56 per cent. Eosinophiles numerous, and poikilocytosis very pronounced. During the course of treatment a day or two had to be omitted owing to high temperature and other symptoms of intoxication occurring on several occasions. After three weeks' treatment the spleen began progressively to decrease in size. The first decided change noticed was that the myelocytes and eosinophiles gradually disappeared, and the erythrocytes returned to their normal shape. By April 12th myelocytes had disappeared, and eosinophiles and poikilocytes scanty. By June patient was able to return to work in excellent health, menstruation, which had been suspended, returned normally, and the last blood examination was normal. Senn has seen no good result in malignant disease; he considers that this case proves that leucæmia is due to a microbic cause, and that the x-rays exert an antimicrobic action.

1 Quarterly Med. Jour., Aug 1903. 2 Brit. Med. Jour., Oct. 24, 1903. 3 Scottish Med. and Surg. Jour., Feb. 1904. 4 Brit. Med. Jour., Oct. 24. 5 Lancet, Jan. 16, 1904. 6 Med. Chron., Dec. 7 Med. Rec., Aug. 22, 1903.

*(To be continued.)*

